# Sociodemographic and Clinical Profiles of Patients With Substance Use Disorders Admitted to a Psychiatric Hospital in Erbil: A Retrospective Study

**DOI:** 10.7759/cureus.75483

**Published:** 2024-12-10

**Authors:** Banaz Saeed

**Affiliations:** 1 Department of Psychiatry, College of Medicine, Hawler Medical University, Erbil, IRQ

**Keywords:** aggression, methamphetamine, psychiatric comorbidity, sociodemographic factors, substance use disorder

## Abstract

Background: Substance use is a growing concern, impacting the health, social stability, and economic well-being of individuals and communities. In Iraq, particularly in Erbil, limited data exists on the sociodemographic and clinical characteristics of patients with substance use disorders (SUDs). This study aims to identify these characteristics among inpatients at Hawler Psychiatric Hospital to better understand the profiles and associated factors influencing substance use in this region.

Methods: A retrospective study was conducted at Hawler Psychiatric Hospital from January 2023 to September 2024. A total of 115 patient records with substance use disorder diagnoses were reviewed. Data on sociodemographic details, clinical characteristics, types of substances used, and comorbidities were collected and analyzed using Microsoft Excel 2016 (Microsoft Corp., Redmond, WA) and IBM SPSS Statistics version 26 (IBM Corp., Armonk, NY). Frequency, percentages, and mean values were calculated, while Chi-square and Fisher’s exact tests were used to assess associations between categorical variables. A P-value of 0.05 or less was deemed statistically significant.

Results: The majority of the patients were male (112, 97.4%), with an age of 31.95 ± 9.46 years (mean ± standard deviation (SD)). Among them, 67 (58.3%) had only primary school education. Methamphetamine was the most commonly used substance (52, 45.2%), followed by alcohol (28, 24.3%), opioids (13, 11.3%), and heroin (13, 11.3%). High rates of psychiatric comorbidities were observed, with 97 (84.3%) patients diagnosed with additional mental health conditions, such as depression and psychosis. Furthermore, 73 (63.5%) patients exhibited aggressive behaviors, and 33 (28.7%) had a forensic history. Significant associations were identified between aggression and factors such as educational level, occupation, psychiatric comorbidity, and forensic history.

Conclusion: The study highlights a pattern of poly-substance use among patients and the pressing need for comprehensive treatment strategies, including both pharmacotherapy and psychotherapy, to address the complex needs of individuals with substance use disorders in Erbil. These findings underscore the importance of targeted interventions and preventive measures to reduce substance misuse and improve patient outcomes.

## Introduction

Drug use and abuse remain a very real problem in many parts of the world, although the extent of the issue varies greatly depending on culture, access, and legislation. In 2021, one in every 17 people aged 15-64 in the world had used a drug in the past 12 months. The estimated number of users grew from 240 million in 2011 to 296 million in 2021 (5.8% of the global population aged 15-64) [1,2].

The impact of substance use disorders (SUDs) on societies in terms of health and mortality, economics, and crime is profound and appears to be worsening [3]. United Nations Office on Drugs and Crime (UNODC) research has found that Iraq is at risk of becoming an increasingly significant node in the drug trafficking ecosystem spanning the Near and Middle East. Within Iraq, drugs are trafficked along three key internal corridors, in the north, central, and southern regions of the country. The main categories of drugs trafficked through Iraq include opium, heroin, hashish, and, especially, methamphetamine and “captagon”. While Iraq is not necessarily the most affected country in the region in terms of volumes of drugs seized, there are risks that the situation could deteriorate if drug trafficking, in particular of methamphetamine and “captagon”, keeps intensifying [4].

The sociodemographic determinants identified, such as age, male gender, urban residence, family history, and lower socioeconomic status, provide valuable insights for developing targeted prevention and intervention strategies. These determinants reflect findings from previous research, emphasizing the need for multifaceted approaches that consider social, economic, and cultural factors influencing substance use [5].

While the incidence of substance use is rising, there appears to be a lack of research on the sociodemographic and clinical profile of patients with substance use disorder in the Erbil region. Therefore, this study aims to assess the sociodemographic and clinical profile of inpatients admitted to Hawler Psychiatric Hospital during 2023-2024. Erbil is the capital and most populous city of the Kurdistan region, located in the northern part of Iraq, with a population of 1.5 million.

## Materials and methods

For this purpose, a retrospective study was conducted at Hawler Psychiatric Teaching Hospital in Erbil, Iraq. The study was conducted over four months, from May 1, 2024, to September 1, 2024.

After prior ethical and institutional permission, a record-based analysis from January 2023 to September 2024 was performed to study the sociodemographic and clinical profiles of inpatients undergoing management at Hawler Teaching Psychiatric Hospital. Patients admitted to the hospital with a diagnosis of substance use disorder during the specified period were included in the study. All patients were indigenous people. Records that lacked necessary information or contained incomplete data were excluded from the research, resulting in the exclusion of five records from the initial total of 120 identified cases.

The questionnaire used for this study was developed by the researchers. It consisted of two main sections: the sociodemographic profile and the clinical profile of inpatients with substance use disorder. The sociodemographic profile included age, gender, marital status, number of children, educational level, occupation, religion, and ethnic background. The clinical profile included the main type of substances used, reason for admission, duration of stay in the hospital, previous history of admission, past medical and surgical history, forensic history, risks of suicide and aggression, history of comorbid medical and psychiatric disorders at the time of admission, and type of treatment administered at the time of admission, whether pharmacological or psychological.

The questionnaire was reviewed for clarity and relevance by applying it to a small sample of five randomly selected medical records prior to full implementation. This process ensured that all necessary data could be reliably extracted and that the questionnaire was suitable for the study objectives.

Ethical approval was obtained from the Research Ethics Committee of the College of Medicine at Hawler Medical University. Permission was obtained from the hospital administration before we began the questionnaire.

The collected data was first entered using Microsoft Office Excel 2016 (Microsoft Corp., Redmond, WA) and then exported to IBM SPSS Statistics for Windows version 26 (IBM Corp., Armonk, NY) for management and analysis. Frequency and percentages were calculated to describe categorical data, while the mean was used for continuous variables, such as age, when necessary. Chi-square and Fisher’s exact tests were applied to assess the significance of associations between categorical variables. A P-value of 0.05 or less was considered statistically significant.

## Results

This study included all 115 files of patients admitted to the psychiatry hospital over the last two years due to substance use disorders. The majority of patients were male (97.4%). The mean age of the patients was 31.95 ± 9.46 years, with most individuals falling within the 20- to 29-year age group (40.9%). The mean age of starting substance use was 26.77 ± 8.54 years, with 46.1% beginning between 20 and 29 years. In terms of marital status, 57.4% were married, and 46.1% had no children. Educationally, 58.3% had completed primary school, while 42.6% were unemployed. Most patients were Kurdish (91.3%) and Muslim (95.7%). Furthermore, 21.7% had undergone previous surgeries, and 28.7% had a forensic history. Regarding medical history, 24.3% of patients had other medical conditions. The most common comorbidities included fatty liver (25%), followed by epilepsy (14.3%) and hypertension (14.3%). Other conditions such as renal disease (10.7%), heart disease (7.1%), and stroke (7.1%) were also reported, with 17.9% having various other medical issues (Table 1).

**Table 1:** Sociodemographic Characteristics of the Patients SD: standard deviation

Variable	Number	%
Gender		
Male	112	97.4
Female	3	2.6
Age (mean ± SD: 31.95 ± 9.46)		
<20	8	7.0
20-29	47	40.9
30-39	32	27.8
40-49	23	20.0
50 and above	5	4.3
Age of starting substance use (mean ± SD: 26.77 ± 8.53)		
<20	21	18.3
20-29	53	46.1
30-39	22	19.1
40-49	8	7.0
50 and above	1	0.9
Missing data	10	8.7
Marital status		
Single	46	40.0
Married	66	57.4
Divorced/widowed	3	2.6
Number of children		
0	53	46.1
1	7	6.1
2	16	13.9
3	23	20.0
4	11	9.6
5	3	2.6
6	2	1.7
Education		
Illiterate	4	3.5
Primary school	67	58.3
Secondary school	31	27.0
College/institute	13	11.3
Occupation		
Employed (manual labors, worker)	47	40.9
Employed (self-employed, business owners)	8	7.0
Employed (skilled, professional)	5	4.3
Military personnel	6	5.2
Unemployed	49	42.6
Ethnicity		
Kurd	105	91.3
Arab	10	8.7
Religion		
Muslim	110	95.7
Christian	5	4.3
Past surgical history		
Present	25	21.7
No surgeries done	89	77.4
Missing data	1	0.9
Medical history		
Presence of other medical conditions	28	24.3
No other medical conditions	87	75.7
Forensic history		
Present	33	28.7
No	82	71.2
Total	115	100.0

The majority of patients in this study were referred from private clinics (84.3%), with smaller numbers coming from hospital outpatient services (14.8%) and the hospital committee (0.9%). The primary reason for admission was detoxification, accounting for 87.8% of cases, followed by aggression (5.2%), psychosis management (4.3%), and other reasons (2.7%). Regarding hospital stay duration, 46.1% of patients stayed between one and five days, while 40.0% stayed for 6-10 days. A smaller percentage stayed for 11-15 days (9.6%) or more than 16 days (2.6%). Two (1.7%) cases had missing data on hospital stay duration (Table 2).

**Table 2:** Referral Sources, Reasons for Admission, and Hospital Stay Duration

Variable	Number	%
Source of referral		
Private clinic	97	84.3
Hospital outpatient	17	14.8
Hospital committee	1	0.9
Reason for referral		
Detoxification	101	87.8
Aggression	6	5.2
Psychosis management	5	4.3
Others	3	2.7
Duration of stay at the hospital (days)		
1-5	53	46.1
6-10	46	40.0
11-15	11	9.6
16 and above	3	2.6
Missing data	2	1.7
Total	115	100.0

The patterns of substance use among the patients revealed that 45.2% were using methamphetamine, making it the most commonly used substance. This was followed by alcohol (24.3%), opioids (11.3%), heroin (11.3%), and smaller percentages using benzodiazepines, cannabis, and pregabalin (2.6% each). Additionally, 89.6% of patients reported using other substances alongside their primary drug. The duration of substance use among participants varied, with 39.1% using substances for 1-4 years. Furthermore, 26.1% reported use for 4-7 years, while 8.7% had used substances for less than one year and 11.3% for 7-10 years. Only 5.2% indicated use for over 10 years, and 9.6% had missing data on their duration of use (Table 3).

**Table 3:** Pattern of Substance Use

Variable	Number	%
Type of substance used		
Methamphetamine	52	45.2
Alcohol	28	24.3
Opioids	13	11.3
Heroin	13	11.3
Benzodiazepines	3	2.6
Cannabis (marijuana)	3	2.6
Pregabalin	3	2.6
Duration of use		
Less than one year	10	8.7
1-4 years	45	39.1
4-7 years	30	26.1
7-10 years	13	11.3
Above 10 years	6	5.2
Missing data	11	9.6
Used other substances? (Poly-substance user or not?)		
Yes	103	89.6
No	12	10.4
Total	115	100.0

**Figure 1:**
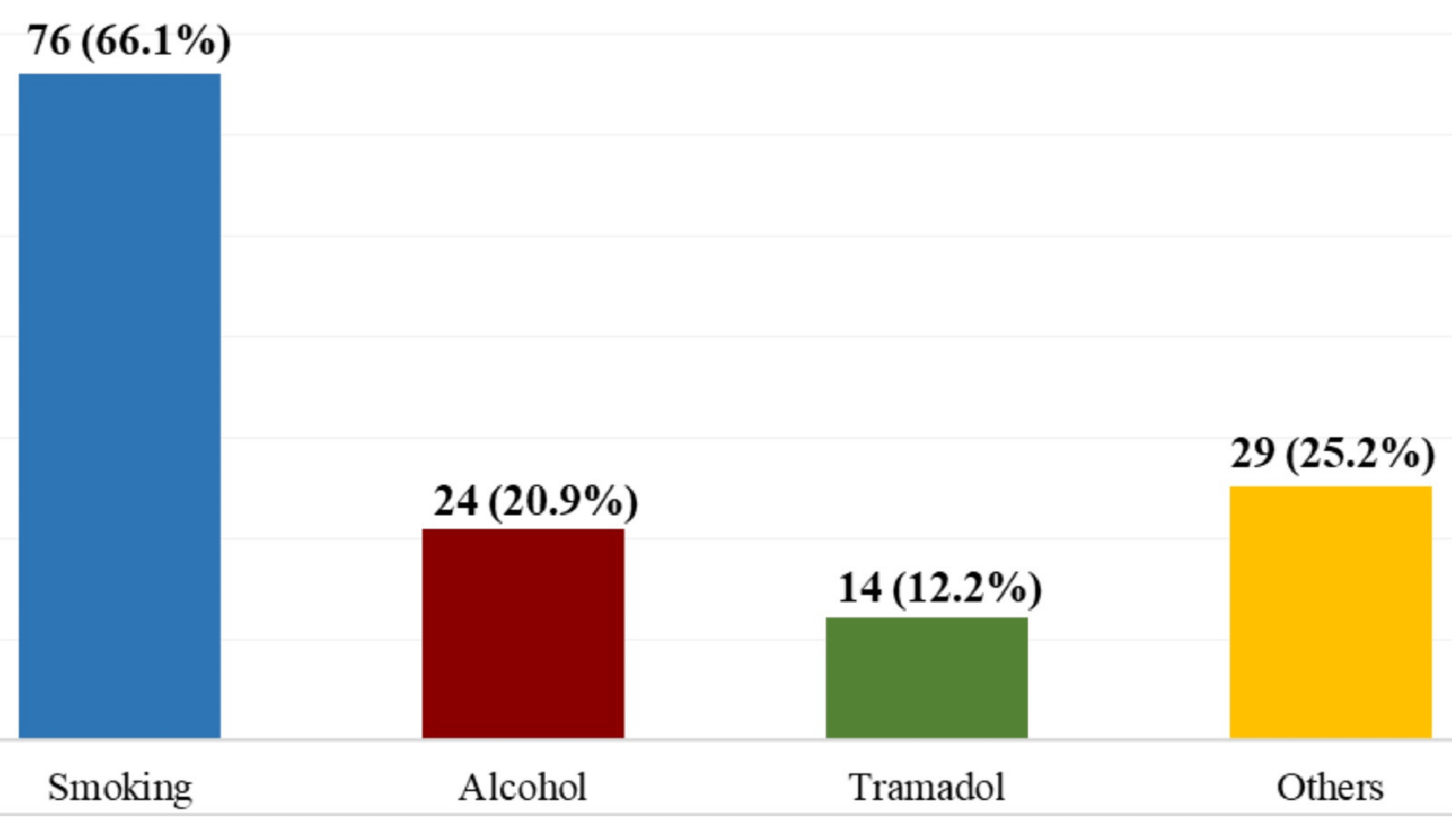
Distribution of Secondary Substances Used Among Patients With Substance Use Disorder (N=115)

Smoking was the most common secondary substance, with 66.1% of patients engaging in it. Other substances included alcohol (20.9%), tramadol (12.2%), and a range of others (25.2%). These findings highlight the polydrug use common among patients admitted for substance-related disorders (Figure 1).

In this study, 63.5% of patients exhibited aggressive behavior, while 36.5% did not. Psychiatric comorbidities were highly prevalent, with 84.3% of patients having at least one additional mental health condition alongside substance use (Table 4).

**Table 4:** Aggression and Psychiatric Comorbidities

Variable	Number	%
Aggression		
Yes	73	63.5
No	42	36.5
Presence of psychiatric comorbidity		
Yes	97	84.3
No	18	15.7
Total	115	100.0

**Figure 2:**
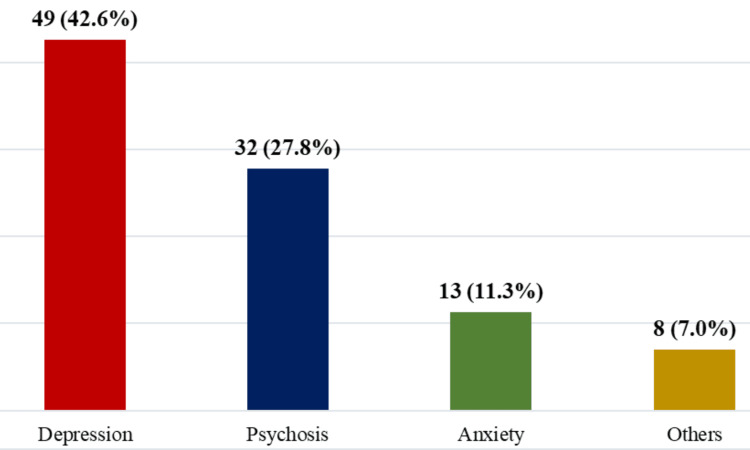
Prevalence of Psychiatric Comorbidities Among Patients With Substance Use Disorder (N=115)

Among those with psychiatric comorbidities, the most common diagnosis was depression, affecting 42.6% of patients, followed by psychosis (27.8%) and anxiety disorders (11.3%). Other psychiatric conditions were present in 7.0% of cases. This data underscores the significant overlap between substance use and other mental health disorders in this population (Figure 2).

In terms of suicide risk assessment, only 27.0% of patients were evaluated for suicide risk, while 73.0% were not assessed. Nearly all patients (99.1%) received medication during their stay, with just one (0.9%) patient not receiving any. However, psychotherapy interventions were rare, with only 2.6% of patients receiving therapy, while the overwhelming majority (97.4%) did not. Additionally, 14.8% of patients had a previous admission to the hospital due to substance use, while 85.2% were being treated for the first time. This suggests a strong focus on pharmacological treatment, with limited use of psychotherapy in managing substance use disorders in this setting (Table 5).

**Table 5:** Suicide Risk Assessment, Medications Administered, and Psychotherapy Interventions

Variable	Number	%
Risk of suicide		
Yes	31	27.0
No	84	73.0
Pharmacotherapy received during admission		
Yes	114	99.1
No	1	0.9
Psychotherapy received after admission		
Yes	3	2.6
No	112	97.4
Previous admission to hospital		
Yes	17	14.8
No	98	85.2
Total	115	100.0

In terms of aggression, the results show that aggression levels vary significantly by education and occupation. All illiterate individuals (100.0%) exhibited aggressive behavior, compared to lower aggression rates in those with higher educational levels, such as college graduates (30.8%). Occupational differences were also notable, with military personnel (83.3%) and the unemployed (71.4%) displaying higher aggression rates than skilled professionals (20.0%) and self-employed individuals (25.0%). The presence of comorbid psychiatric disorders significantly influenced aggression, with 69.1% of individuals with psychiatric disorders exhibiting aggressive behavior, while only 33.3% of those without psychiatric disorders were aggressive. Additionally, forensic history was a strong predictor of aggression, with 84.8% of those with a criminal record showing aggressive behavior compared to 54.9% of those without a record (Table 6).

**Table 6:** Association Between Aggression and Other Variables Statistical analysis was performed using the Chi-square test. The symbol * indicates values calculated using Fisher’s exact test due to small sample sizes in specific categories.

Variable	Aggression		P-value
	Yes	No	
	Number (%)	Number (%)	
Education			0.033*
Illiterate	4 (100.0)	0 (0.0)	
Primary school	45 (67.2)	22 (32.8)	
Secondary school	20 (64.5)	11 (35.5)	
College/institute	4 (30.8)	9 (69.2)	
Occupation			0.021*
Manual labor, workers	30 (63.8)	17 (36.2)	
Self-employed, business owners	2 (25.0)	6 (75.0)	
Skilled, professional	1 (20.0)	4 (80.0)	
Military personnel	5 (83.3)	1 (16.7)	
Unemployed	35 (71.4)	14 (28.6)	
Reason of referral			0.077*
Aggression	6 (100.0)	0 (0.0)	
Detoxification	60 (59.4)	41 (40.6)	
Psychosis management	5 (100.0)	0 (0.0)	
Others	2 (66.7)	1 (33.3)	
Type of substance			0.075*
Alcohol	14 (50.0)	14 (50.0)	
Heroin	6 (46.2)	7 (53.8)	
Methamphetamine	38 (73.1)	14 (26.9)	
Tramadol	7 (63.6)	4 (36.4)	
Others	8 (72.7)	3 (27.3)	
Comorbid psychiatric disorder			0.004
Yes	67 (69.1)	30 (30.9)	
No	6 (33.3)	12 (66.7)	
Forensic history			0.003
Yes	28 (84.8)	5 (15.2)	
No	45 (54.9)	37 (45.1)	

## Discussion

This study represents the first regional investigation into the sociodemographic and clinical profiles of patients admitted to the psychiatric ward with a history of substance use disorder in Erbil. The present study was conducted among 115 patients with substance use, aiming to investigate various sociodemographic and clinical aspects of substance use in a psychiatric hospital setting.

In the present study, men (97.4%) were the predominant substance users, consistent with findings from other studies [6,7], which have also reported that substance use is more prevalent among men. Furthermore, as highlighted in previous household surveys, Iraqi women report significantly lower rates of substance use compared to Iraqi men. This disparity may be attributed to cultural and gender norms [8].

More than half (57.4%) of the patients in the present study were married, while 42% were either unmarried or divorced. These findings align with a previous study [9]. A possible explanation could be that the pressures associated with marriage, careers, and raising children in our culture may increase the risk of substance abuse, despite the potential for marriage to provide a supportive and structured environment. However, our results contrast with two other studies that reported a lower prevalence of substance use among married men [10,11].

Substance users in this study were predominantly individuals with a primary school education. These findings are consistent with a previous study in Punjab [12], which also highlighted a lower educational level among substance users. Limited education may result in reduced awareness of the harmful effects of substance use and fewer employment opportunities, potentially increasing stress and fostering an environment conducive to continued substance use.

The majority of the sample studied were either unemployed or employed in low-paying jobs, indicating that they were in a poor economic situation within the city. These results are consistent with previous studies [13,14], which found that substance use is more prevalent among the unemployed.

Despite results from previous studies concluding that Muslims with high religiosity report less alcohol and substance use as a protective factor [15-17], 95.7% of the studied sample were Muslim, as Islam is the predominant religion in the city. At the same time, the global supply of opium is currently produced and trafficked through an extensive network of countries that span Southeast and Central Asia, especially Afghanistan, Iran, and Pakistan. The result is that these countries have a high prevalence of opiate use and injection drug use [18]. This raises the importance of the issue that substance use is increasing in Muslim countries.

In this study, 28.7% of the studied sample had a forensic history. It is well known from previous studies [19-22] that substance use disorders (SUDs) are more prevalent in forensic populations than in the non-forensic or general population, and having an SUD is a known risk factor that leads patients to enter forensic services [23-26].

The study revealed that 84.3% of the group was referred from private specialist clinics to the hospital, while only 14.8% were referred from outpatient clinics. This finding suggests that either patients with substance use disorders are not visiting outpatient clinics or they are not disclosing their substance use during these visits. This indicates that fear or stigma surrounding substance use remains prevalent in the area. Previous studies suggest that the stigma toward substance use disorders surpasses that of other mental health conditions and is a common barrier to help-seeking behavior among individuals with SUDs [27,28].

Among the studied sample, 87.8% were referred to the hospital for detoxification. However, nearly half of them stayed for fewer than five days. At the same time, the majority of the study sample used the substance for more than one year, indicating that they needed a longer stay in the hospital. This was inconsistent with a previous study from New York [29], which concluded that individuals with substance use disorders had longer hospital stays. The finding that nearly 46% of patients with substance use disorders (SUDs) stayed in the hospital for fewer than five days highlights a significant trend in inpatient care. Prior studies indicate an average stay of 5-10 days for SUD-related hospitalizations, with 6.4 days being common in the US healthcare system [30]. Shorter stays may reflect a focus on acute stabilization rather than comprehensive treatment, which risks higher relapse rates if not followed by robust outpatient care. Comparatively, longer stays have been associated with better outcomes in settings that prioritize detoxification and therapy integration.

Methamphetamine was the most common substance among the studied group, with 45.2% reporting it as their primary substance of use. This result is consistent with previous studies that conclude that the prevalence of methamphetamine use has been steadily increasing globally, coinciding with similar increases in methamphetamine use disorder and other associated harms [31-34]. The quick rise in methamphetamine use is a concerning public health problem, as methamphetamine is a potent stimulant that can have significant effects on both physical and mental health [35]. The second most common substance was alcohol, with 24.3% of participants reporting its use. This was consistent with previous studies conducted in Turkey [36], where 20.3% reported alcohol use. Opioid use was the third most common substance among the studied group, with 22.7% using different types of opium, including heroin, tramadol, and other opium. This was slightly less than other studies done in Iran, Punjab, and Kashmir [37-39].

In this study, 89% of the participants were poly-substance users, consuming multiple types of substances. This was consistent with a previous study done in Iraq and Vindhya region [40,41]. This finding highlights the seriousness of their condition and suggests significant challenges for treatment. Smoking was the most common second substance, reported by 66% of these patients. These results are in line with studies done in Norway, Australia, and Germany [42-44].

The finding that 42.6% of individuals used a substance for more than four years aligns with studies that emphasize the persistence of substance use over long periods, especially among those who begin use at a young age. For instance, a study by Volkow et al. found that long-term substance use was particularly common among youth who began using before adulthood [45]. These individuals often showed a tendency for substance use to persist over several years, significantly increasing the risk of dependency and related disorders.

Another study by Capella et al. analyzed adult males with long-term substance use histories. This study reported a similar trend, where a substantial percentage of participants had been using substances for several years, often more than four [46]. Comparing these studies with the current findings shows a consistent pattern; a significant proportion of individuals engaging in substance use continue beyond the four-year mark, especially those with earlier onset. These findings emphasize the persistent nature of substance use and its risk factors, highlighting the importance of early intervention and long-term treatment strategies.

The risk of aggression was high among the studied group, with 63% experiencing aggression during their substance use. This result aligns with previous studies [47-49], which concluded that rates of aggression and violence are often elevated among substance users.

The study revealed that 84% of the studied group exhibited symptoms of other psychiatric disorders, such as depression, psychosis, and anxiety at the time of admission. The European Monitoring Centre for Drugs and Drug Addiction (EMCDDA) reports a high prevalence of psychiatric comorbidities among substance users, particularly mood and anxiety disorders [50].

The risk of suicide among admitted patients with substance use disorder was 27%. Lynch et al. [51] examined the association between substance use disorders and suicide risk in a systematic review. They noted that individuals with substance use disorders have a significantly elevated risk of suicidal thoughts and behaviors, emphasizing the need for targeted interventions in this population.

The majority of cases were admitted to the hospital for the first time, and they received pharmacotherapy upon admission for detoxification, with only 2.6% being referred for psychotherapy or rehabilitation. Previous systematic reviews [52] have highlighted that integrating both pharmacotherapy and psychotherapy leads to improved outcomes compared to traditional care. This discrepancy is likely due to the limited availability of psychotherapy and rehabilitation services within the hospital setting.

This study has limitations due to its retrospective design, primarily the reliance on existing hospital records, which may not always be complete or accurate. The clinical profile of the patients was assessed using an ad hoc questionnaire designed specifically for this study to capture data from hospital records. While this tool allowed for the collection of context-specific information, it lacks psychometric validation. Additionally, we could not assess the severity of patients’ symptoms or obtain current data on the social and economic state of the studied group. Despite these limitations, this study addresses an important research gap by identifying factors associated with psychiatric admissions in Erbil. Future studies should integrate validated instruments, either by adapting existing tools to the local context or supplementing them with hospital-specific variables, to improve methodological rigor and ensure comparability with broader research.

## Conclusions

This study highlights the significant public health concern of substance use disorders (SUDs) among admitted patients at Hawler Psychiatric Hospital in Erbil. Methamphetamine was identified as the most commonly used substance, followed by opioids and alcohol. The study reveals a high prevalence of psychiatric comorbidities, including depression and anxiety, which, along with the elevated risk of aggression and suicide, underscores the need for integrated treatment approaches. The study also revealed that there is a significant association between aggression and educational level, occupation, comorbid psychiatric disorder, and forensic history. A comprehensive approach that combines pharmacotherapy and psychotherapy is essential to improve outcomes for this vulnerable population. Early intervention, targeted prevention, and a better understanding of the associated risks are vital to promoting healthier outcomes for both individuals and the broader community.

Based on the findings, comprehensive treatment programs that combine pharmacotherapy and psychotherapy are critical for addressing the complex needs of patients admitted with substance use disorders and psychiatric comorbidities in Erbil. Targeted prevention efforts should focus on educational initiatives to reduce substance misuse, particularly among those with lower educational levels. The high prevalence of aggressive behaviors highlights the need for specialized management programs, particularly for patients with forensic histories or mental health conditions. Additionally, involving family members and the community in treatment could enhance recovery and reduce the risk of relapse.

## Appendices

Sociodemographic factors

1. Age: ______ (years)

2. Gender

a. Male

b. Female

3. Marital state

a. Single

b. Married

c. Divorced

4. Number of children: _____

5. Educational level

a. Illiterate

b. Primary school

c. Secondary school

d. College/institute

e. Postgraduate

6. Employment

a. Employed (manual labor, workers)

b. Employed (self-employed, business owners)

c. Employed (skilled, professional)

d. Military personnel

e. Unemployed

7. Ethnicity

a. Kurdish

b. Arabic

c. Others

8. Religion

a. Muslim

b. Christian

c. Others

Clinical factors

9. Source of referral

a. Private clinic

b. Psychiatric outpatient

c. Committees

10. Reason for referral

a. Detoxification

b. Psychiatric treatment

c. Others (specify: ___________________________)

11. Duration of stay at the hospital: _______ days

12. Type of substance that the patient admitted due to

a. Methamphetamine

b. Alcohol

c. Opioids

d. Heroin

e. Benzodiazepines

f. Cannabis (marijuana)

g. Pregabalin

h. Others (please specify: ___________________)

13. Patient uses other substances

a. Yes (please specify: ____________________________)

b. No

14. Duration of substance use

a. Less than 1 year

b. 1-4 years

c. 4-7 years

d. 7-10 years

e. More than 10 years

15. Age of starting to use substance: ______ years

16. Previous history of admission to psychiatric ward

a. Yes

b. No

17. Past medical history

a. Yes (If yes, please specify: ________________________)

b. No

18. Past surgical history

a. Yes

b. No

19. Forensic history

a. Yes

b. No

20. Risk of suicide at the time of admission

a. Yes

b. No

21. Aggression at the time of admission

a. Present

b. No

22. Comorbid psychiatric problems

a. Present (If so, please specify: ___________________)

b. No

23. Type of treatment received as a plan of management

a. Pharmacotherapy

b. Psychotherapy

c. Both
